# Supplementary material for: Physiological and Transcriptomic Analyses Reveal the Effects of Carbon-Ion Beam on Taraxacum kok-saghyz Rodin Adventitious Buds
Source: Int J Mol Sci. 2023 May 26;24(11):9287. doi: 10.3390/ijms24119287 (PMC10252833; doi:10.3390/ijms24119287)
Supplement: Supplementary file 1 [file ijms-24-09287-s001.zip › ijms-2304966-supplementary.pdf]

Supplementary Table

Table S1 Quality control of sequencing data

Sample	Total Clean Reads (M)	Clean bases (Gb)	GC Content (%)	≥ Q30（%）	Uniquely Mapping (%)
C02_1	54.94	8,22	44.82	93.46	79.07
C02_2	54.92	8,22	44.91	93.56	78.85
C02_3	55.55	8,30	45.01	93.78	79.03
C402_1	51.83	7,75	44.49	93.81	78.85
C402_2	51.18	7,66	44.93	93.44	78.59
C402_3	48.42	7,24	44.84	93.63	78.65
C06_1	55.22	8,24	45.00	93.40	78.67
C06_2	56.84	8,49	45.04	93.88	79.33
C06_3	53.73	8,02	45.24	93.95	79.51
C406_1	63.61	9,53	44.37	93.32	78.46
C406_2	43.85	6,55	44.52	94.12	78.80
C406_3	67.39	10,10	44.80	93.33	76.16
C024_1	48.67	7,29	44.88	93.66	79.42
C024_2	46.12	6,91	44.77	94.01	78.82
C024_3	53.56	8,03	44.60	93.94	78.80
C4024_1	49.26	7,37	43.78	93.42	76.96
C4024_2	52.84	7,10	44.89	93.18	76.64
C4024_3	54.56	8,17	44.24	93.30	78.13
C072_1	54.08	8,09	45.45	93.98	71.90
C072_2	52.62	7,87	44.31	93.48	77.20
C072_3	54.85	8,21	44.54	93.60	79.09
C4072_1	48.95	7,33	44.22	93.65	76.51
C4072_2	43.21	6,47	44.75	93.97	77.69
C4072_3	45.42	6,80	44.31	94.04	78.60
